# Young aboriginals are less likely to receive a renal transplant: a Canadian national study

**DOI:** 10.1186/1471-2369-14-11

**Published:** 2013-01-14

**Authors:** Steven Promislow, Brenda Hemmelgarn, Claudio Rigatto, Navdeep Tangri, Paul Komenda, Leroy Storsley, Karen Yeates, Julie Mojica, Manish M Sood

**Affiliations:** 1 Department of Medicine, Section of Nephrology, St Boniface Hospital, University of Manitoba, 409 Tache Avenue, Winnipeg, R2H 2A6, Canada; 2 Department of Medicine, Section of Nephrology, Foothills Hospital, University of Calgary, 1403 29 Street, Calgary, AL, T2N2T9, Canada; 3 Department of Medicine, Section of Nephrology, Seven Oaks Hospital, University of Manitoba, 2300 McPhillips Street, Winnipeg, R2V 3M3, Canada; 4 Department of Medicine, Section of Nephrology, Health Sciences Centre, University of Manitoba, 820 Sherbrook street, Winnipeg, MB, R3A 1R9, Canada; 5 Department of Medicine, Section of Nephrology, Kingston General Hospital, Queen’s University, 94 Stuart Street, Kingston, ON, K7L2V6, Canada

## Abstract

**Background:**

Previous studies have demonstrated Aboriginals are less likely to receive a renal transplant in comparison to Caucasians however whether this applies to the entire population or specific subsets remains unclear. We examined the effect of age on renal transplantation in Aboriginals.

**Methods:**

Data on 30,688 dialysis (Aboriginal 2,361, Caucasian 28, 327) patients obtained between Jan. 2000 and Dec. 2009 were included in the final analysis. Racial status was self-reported. Cox proportional hazards, the Fine and Grey sub-distribution method and Poisson regression were used to determine the association between race, age and transplantation.

**Results:**

In comparison to Caucasians, Aboriginals were less likely to receive a renal transplant (Adjusted HR 0.66 95% CI 0.57-0.77, P < 0.0001) however after stratification by age and treating death as a competing outcome, the effect was more predominant in younger Aboriginals (Age 18–40: 20.6% aboriginals vs. 48.3% Caucasians transplanted; aHR 0.50(0.39-0.61), p < 0.0001, Age 41–50: 10.2% aboriginals vs. 33.9% Caucasians transplanted; aHR 0.46(0.32-0.64), p = 0.005, Age 51–60: 8.2% aboriginals vs. 19.5% Caucasians transplanted; aHR0.65(0.49-0.88), p = 0.01, Age >60: 2.7% aboriginals vs. 2.6% Caucasians transplanted; aHR 1.21(0.76-1.91), P = 0.4, Age X race interaction p < 0.0001). Both living and deceased donor transplants were lower in Aboriginals under the age of 60 compared to Caucasians.

**Conclusion:**

Younger Aboriginals are less likely to receive a renal transplant compared to their Caucasian counterparts, even after adjustment for comorbidity. Determination of the reasons behind these discrepancies and interventions specifically targeting the Aboriginal population are warranted.

## Background

While the number of Aboriginal patients with end-stage renal disease (ESRD) requiring dialysis in Canada has increased rapidly over the last few decades, a similar growth in renal transplantation rates for this population has not occurred [1]. Transplantation is the ideal therapeutic option for the ESRD population with a significant survival advantage, decreased morbidity and cost, and improved quality of life [2]. The Aboriginal ESRD population is in general younger than their Caucasian counterparts and often reside in rural communities [3]. Previous studies have shown, ESRD for the Aboriginal population on dialysis typically requires displacement from their communities and disruption of their lifestyle [4].

Despite the fact that the Aboriginal population with ESRD seem well suited for renal transplantation, they continue to receive transplants at a significantly lower rate compared to their Caucasian counterparts [5]. Decreased rates of both living and deceased donor transplants have been documented in various regions across Canada, and the largest international study to date indicates Aboriginals receive transplants at roughly half the rate compared to the Caucasian ESRD population [6,7]. There are many theories that have been postulated as to why this discrepancy exists such as a decrease in living donors, residing in remote communities or low socioeconomic status but to date no clear reasons have been brought to the forefront. In renal transplantation, age has been found to be an important effect modifier with lower rates of renal transplantation demonstrated in elderly women [8].

With this in mind, we set out to examine age as an effect modifier on the rates of renal transplantation in Aboriginal compared to the Caucasian population.

## Methods

### Study design

All adults (>18 years old) who received dialysis therapies or a renal transplantation from the Canadian Organ Replacement Registry (CORR) between January 1, 2000 and December 2009 were included in our analysis. In patients who received greater than one renal transplant, only the first transplant was included in our analysis. The Canadian CORR captures data on all dialysis patients in Canada (except Quebec) and includes information on demographics, death, dialysis modality, comorbidities and transplantation. CORR data has been well validated for the study of outcomes [9]. This study was reviewed by the Research Board and the Hospital Ethics Board at St. Boniface Hospital in Winnipeg, Manitoba. All data was de-identified, retrospective from the CORR.

### Definitions

Racial information is recorded by health care providers based on patient self report. Dialysis modality was determined 90 days after dialysis initiation and categorized as hemodialysis or peritoneal dialysis. Co-morbid illnesses included a history of angina, myocardial infarction, diabetes mellitus, peripheral vascular disease, malignancy, hypertension medication usage, cigarette smoker, lung disease and stroke. Causes of ESRD included ischemia, diabetes mellitus, glomerulonephritis, interstitial disease, polycystic kidney disease, obstruction, other and unknown. Provinces and territories were categorized as geographic regions as follows: Atlantic (New Brunswick, Nova Scotia, Prince Edward Island, Newfoundland), Central (Ontario), Prairies (Alberta, Saskatchewan, Manitoba, Nunavut, Northwest Territories), Pacific (British Columbia, Yukon). Pre-dialysis care was the length of time from the first Nephrologist visit to the initiation of dialytic therapy. Distance to centre was calculated as the direct linear distance in kilometres between a patients postal code from their primary residence at dialysis initiation to the nearest dialysis provider using Vincenty’s formula [10]. Comorbidities and laboratory data were ascertained at the onset of ESRD.

### Outcome measures

The outcome of interest was renal transplantation with the competing event of mortality. In patients who received greater than one renal transplant, only the first was included in the analysis.

### Statistical analyses

Continuous variables of interest were summarized as mean or medians with standard deviation or inter-quartile range as appropriate. Differences in baseline characteristics were determined by student’s *t*-test or the Kruskal Wallis test for continuous variables and chi-square or the Mann–Whitney test for dichotomous variables.

To assess our outcome of renal transplantation, we utilized traditional Cox proportional hazards and the modified risks regression according to Fine and Gray to account for competing risks [11]. We performed sequential adjustment for demographics, age, sex, race, distance from center, pre-dialysis care, geographic region, comorbidity, BMI, cause of ESRD, serum albumin, and dialysis modality. The competing risk model by Fine and Gray yields an adjusted sub hazard ratio (aSHR). To assess whether age was an effect modifier for renal transplantation, a formal age X race interaction term was examined in crude and adjusted models by both methodologies and found to be statistically significant (p < 0.0001). Based on the distribution of transplantation events, age was further stratified into age groups of 18–40, 41–50, 51–60, >60 years old [12]. As there were a relatively small number of events in the Aboriginal group, additional adjusted models were constructed as follows: model 1 sex, BMI, region, model 2 co-morbidities, and model 3 cause of ESRD, serum albumin, distance from centre, modality, pre-dialysis care.

We determined relative rate ratios (RRR) of total renal transplantation and the subgroups of living or deceased donor organs using Poisson loglinear regression. When all renal transplants were examined, the models were adjusted for similar variables as above. In separate models examining deceased and living donors, models were adjusted for sex, diabetes, vascular disease, region, pre-dialysis care, serum albumin and distance to centre due to a limited number of events.

**Figure 1:**
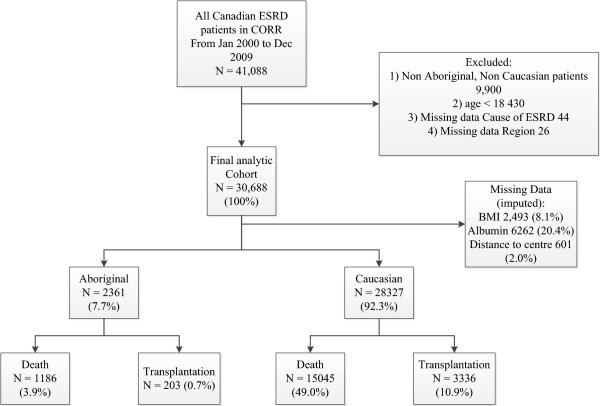
**Development of the study cohort. **ESRD end stage renal disease, CORR Canadian organ Replacement Registry, BMI body mass index.

Multiple imputation was employed for missing values with a random draw from the predictive distribution from an imputation model repeated ten times (see Figure 1). There were no differences in missing data between races. An iterative Markov chain Monte Carlo (MCMC) method was used and pooled estimates of 10 rounds of imputation reported. Models were repeated with original and imputed data to confirm imputation did not significantly alter point estimates.

Analyses were performed using PASW Version 18 and the Fine and Grey analyses were performed using R. All hypothesis tests were two sided with statistical significance to find as having a P value of <0.05.

## Results

Our analytic cohort consisted of 30, 688 [Aboriginal 2,361(7.7%), Caucasian 28,327(92.3%)] patients between Jan 2000 and Dec 2009 in the CORR database. Loss to follow up was minimal (12 Aboriginals and 117 Caucasians) and they were excluded from the analysis. Missing data elements and proportion imputed are presented in Figure 1. The median follow up time for Aboriginals was 2.96 years (IQR 1.51-5.19) and 2.64 years (IQR 1.2-4.86) for Caucasians. Aboriginals were younger (55 vs. 67 mean age), and more likely to be female (50 vs. 40%), with a higher BMI (29.4 vs. 27.8 mean) and reside in rural settings (65.1 vs. 26.5%) than Caucasians. Aboriginals on dialysis reside in the Prairies and they are less likely to have a history of cardiac disease (angina, ACS, pulmonary edema or CABG) or malignancy at dialysis initiation. Conversely they are more likely to have ESRD due to diabetes mellitus and laboratory derangements at dialysis initiation such as anemia, hypoalbuminemia and hyperphosphatemia. Aboriginals were also more likely to initiate dialysis via a central venous catheter and less likely to be on PD 90 days after dialysis initiation (see Table 1).

**Table 1:** Comparison of baseline characteristics among Aboriginals and Caucasians

**Characteristic**			
	**Aboriginal**	**Caucasian**	**P Value**
N	2,361	28,327	
Age (± SD)	55.25 ± 14.1	66.5 ± 14.5	<0.0001
Sex % female (N)	50.0(1180)	39.8(11265)	<0.0001
BMI (± SD)	29.4 ± 6.8	27.8 ± 7.1	<0.0001
Distance to centre in km(IQR)	167.5(217)	12.0 (47)	<0.0001
Rural % (N)	65.1(1531)	26.5(7493)	<0.0001
Median number of days with pre-dialysis care (IQR)	108 (588)	119(790)	0.6
Any pre-dialysis care % (N)	73.7(1739)	71.9(20366)	0.07
Geographic region % (N)			<0.0001
Atlantic	3.0(72)	12.5(3537)	
Central	25.2(594)	54.7(15495)	
Prairie	61.5(1453)	20.6(5847)	
Pacific	10.2(242)	12.2(3448)	
Co-morbidities: % (N)			
Angina	17.4(410)	22.1(6248)	<0.0001
Acute coronary syndrome	17.4(411)	21.9(6195)	<0.0001
Pulmonary edema	30.7(582)	27.9(6177)	0.01
Diabetes mellitus type 1	0.9(21)	0.7(210)	0.4
Diabetes mellitus type 2	70.8(1671)	43.2(12246)	<0.0001
Stroke	12.6(297)	14.3(4049)	0.02
Peripheral vascular disease	21.7(513)	19.5(5516)	0.009
Malignancy	4.5(107)	13.5(3810)	<0.0001
Lung disease	7.9(187)	12(3389)	<0.0001
Hypertension medications	80.3(1896)	80.6(22820)	0.8
Current smoker	23.3(549)	13.4(3803)	<0.0001
CABG	7.9(187)	14(3961)	<0.0001
Cause of ESRD % (N)			
Hypertension	6.6(157)	24(6797)	<0.0001
Diabetes mellitus	64.2(1516)	34.5(9775)	<0.0001
Glomerulonephritis	16(378)	14.2(4021)	0.02
Obstruction	1.3(30)	3.2(913)	<0.0001
Interstitial	0.9(22)	1.1(315)	0.5
Polycystic kidney disease	0.9(22)	4.9(1396)	<0.0001
Other	3.7(88)	8.6(2430)	<0.0001
Unknown	6.3(148)	9.5(2680)	<0.0001
Serum Albumin g/L (± SD)	28.2 ± 6.9	31.8 ± 6.8	<0.0001
Hemoglobin g/L (± SD)	93.5 ± 18.1	100.8 ± 17.2	<0.0001
Phosphorous mmol/L (± SD)	2.21 ± 0.82	1.96 ± 0.73	<0.0001
Peritoneal dialysis % (N)	19.6(463)	21.6(6120)	0.02
AVF/AVG % (N)	8.9(210)	11.0(3130)	0.001

N = cohort size, SD standard deviation, km kilometer, IQR interquartile range, CABG coronary artery bypass graft, ESRD end stage renal disease, g/L grams per litre, mmol/L milimole per litre, AVF arteriovenous fistula, AVG arteriovenous graft.

During the study period, 3,529 (11.5%) received a renal transplant. 203 (8.6%) of Aboriginals with ESRD received a transplant compared to 3,336 (11.7%) of Caucasians. Among Aboriginals age 18–40, only 20.6% underwent renal transplantation compared to 48.3% of Caucasians. This trend was less prominent in the age 51–60 category as 8.2% of Aboriginals were transplanted compared to 19.5% of Caucasians and attenuated in the over 60 age group, albeit with a small number of transplants at 2.7 and 2.6%, respectively. In the time to event analyses differences were observed between the traditional cox models (aHR) and the Fine and Grey method (sHR) accounting for the competing risk of mortality and this effect was more evident in individuals less than 50 (age 18–40: sHR 0.50 95%CI 0.39-0.61, p < 0.0001 vs. aHR 0.62 95%CI 0.49-0.78, p < 0.0001; age 41–50: sHR 0.46 95%CI 0.32-0.64, p < 0.0001 vs. aHR 0.62 95%CI 0.44-0.87, p = 0.005) (see Table 2). Additional models 1 thru 3 yielded point estimates consistent with our fully adjusted models (see Additional file 1: Table S1).

**Table 2:** Crude proportions and adjusted hazard ratio for receiving a renal transplant by age groups in Aboriginals by traditional Cox and competing risk models

**AGE group**	**Proportion transplanted % (N)**		**Competing Risks (95% CI)**	**COX (95% CI)**
	**Aboriginal**	**Caucasian**		
18-40	20.6 (83)	48.3 (1037)	0.50(0.39-0.61), P < 0.0001	0.62(0.49-0.78), p < 0.0001
41-50	10.2 (42)	33.9 (892)	0.46(0.32-0.64), P < 0.0001	0.62(0.44-0.87), p = 0.005
51-60	8.2 (55)	19.5 (899)	0.65(0.49-0.88), P = 0.005	0.68(0.50-0.92), p = 0.01
>60	2.7 (23)	2.6 (508)	1.21(0.76-1.91), P = 0.4	1.22(0.78-1.90), P = 0.4

RACE X Age interaction P < 0.0001 by both COX and competing risks methods.

Across each age category, Caucasians were the referent.

Adjusted for sex, co-morbidity, BMI, albumin, distance, cause of ESRD, PD, pre-dialysis care, region.

HR hazard ratio, CI confidence interval, N cohort size.

**Figure 2:**
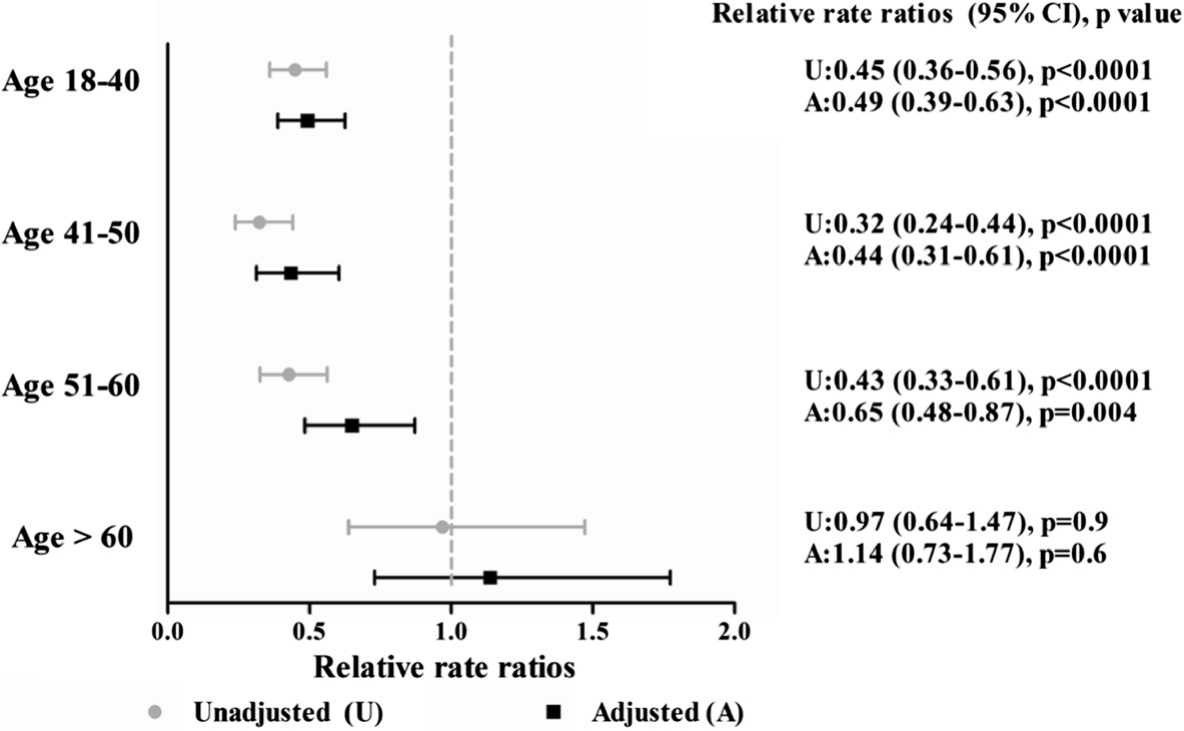
**Unadjusted and adjusted relative rate ratios for renal transplantation in Aboriginals and Caucasians according to age categories. **Adjusted for sex, co-morbidity, BMI, albumin, distance from centre, cause of ESRD, PD, pre-dialysis care, region.

Similar differences were observed when examining crude and adjusted relative rate ratios (RRR) for renal transplantation (see Figure 2). The adjusted relative rate ratio of renal transplantation for Aboriginals age 18–40 was nearly half that of Caucasians (adjusted RRR 0.49 95%CI 0.39-0.63, p < 0.0001) (see Figure 2). This trend was less prominent in the age 51–60 category (adjusted RRR 0.65 95%CI 0.48-0.87, p = 0.004) and again attenuated in the over 60 age group (adjusted RRR 1.14 95%CI 0.73-1.77, p = 0.6).

Information regarding the source of the organ, either living or deceased donor, is presented in Table 3. The adjusted relative rate ratios of living and deceased donor transplants were lower in Aboriginals under the age of 60 compared to Caucasians. This discrepancy was considerably more apparent among living donor transplantation (Age 18–40 adjusted RRR: 0.32 95%CI 0.22-0.47, P < 0.0001) than deceased donors (Age 18–40 adjusted RRR: 0.55 95%CI 0.39-0.76, P < 0.0001).

**Table 3:** Unadjusted and adjusted relative rate ratios rates for living and deceased donor renal transplantation in Aboriginals and Caucasians according to age categories

**AGE group**	**Living donor: Relative risk ratio (95% CI), p value**		**Deceased donor: Relative risk ratio (95% CI), p value**	
	**Crude**	**Adjusted**	**Crude**	**Adjusted**
18-40	0.32(0.22-0.47), p < 0.0001	0.32 (0.22-0.48), p < 0.0001	0.53(0.39-0.72), P < 0.0001	0.55(0.39-0.76), P < 0.0001
41-50	0.21(0.12-0.39), p < 0.0001	0.30(0.16-0.55), P < 0.0001	0.44(0.30-0.63), p < 0.0001	0.50(0.33-0.75), P = 0.001
51-60	0.32(0.19-0.54), p < 0.0001	0.44(0.26-0.76), p = 0.003	0.47(0.33-0.68), P < 0.0001	0.70(0.47-1.03), P = 0.07
>60	0.97(0.46-2.10),p = 0.9	1.05(0.47-2.31), P = 0.9	0.70(0.36-1.36), p = 0.3	0.95(0.47-1.91), P = 0.9

Adjusted for sex, diabetes, vascular disease, region, pre-dialysis care, albumin, distance.

Across each age category, Caucasians were the referent.

CI confidence interval.

## Discussion

In this large, contemporary Canadian cohort study we demonstrated that age is a significant and important factor in receipt of a renal transplantation in Aboriginal peoples. The young Aboriginal ESRD population in particular, the age at which the most benefit is likely to be derived from renal transplantation, are less likely than Caucasian counterparts to receive transplantation. The results of this study, coupled with the rapidly-growing population of young Aboriginals in Canada, suggests that the significantly reduced rate of renal transplantation in the young Aboriginal population may continue to play a significant role in the burden of chronic disease and resultant health challenges they face if some degree of targeted intervention is not undertaken.

Over the last decade, the likelihood of undergoing a renal transplantation is significantly lower in the younger Aboriginal population compared to their Caucasian counterparts. Aboriginals compared to Caucasians between the age of 18 to 40 are less than half as likely to receive a graft (adjusted RRR 0.49 95%CI 0.39-0.63, p < 0.0001). This discrepancy decreases with age as transplant rates become similar in individuals over the age of 60 albeit with few transplants conducted in that age category. This observation remained consistent after adjustment for confounding variables in time to event analyses, after accounting for competing outcomes and when examining ESRD population rates.

Numerous reports have described reductions in renal transplantation in Aboriginal peoples [5,7,13,14]. Earlier reports examining data from the 1990s and early 2000s demonstrated Aboriginals have roughly half the chances of undergoing renal transplantation. Unfortunately our study demonstrates that after a decade little has changed. Despite differing health care delivery systems and ancestral background of Indigenous peoples, reports from Australia, New Zealand and the United states all demonstrate similarly low transplantation rates [5]. Recently, reduced transplant rates have been reported in Canada’s Aboriginal children and adolescents [14]. Although consistent, no studies have illustrated this discrepancy differs with age.

Our analysis included models incorporating competing outcomes allowing the elucidation of important differences in effect estimates. Recently a report of over 1 million ESRD patients in United States, demonstrated reduced survival in African-Americans under the age of 50, largely attributable to the a lower transplantation rate as patients who do not undergo transplant will remain on, and eventually experience mortality on dialytic therapies [15]. This observation was attributed to the use of adjusted models accounting for competing outcomes as in the present analysis. In populations with differential rates of an outcome, traditional Cox models would censor patients; effectively treating them all equally (termed informative censoring). Employing competing risk models yields an effect estimate for transplantation that accounts for mortality differences between the Aboriginal population and Caucasian populations [16,17]. This is illustrated by the differences in the point estimate for transplantation in Aboriginals under the age of 50. With traditional Cox models, the adjusted HR for Aboriginals age 18–40 is 0.62 (95%CI 0.49-0.78) compared to the competing risks adjusted HR of 0.50 (95%CI 0.39-0.61). This is suggestive of mortality differences between the two populations however it should be noted there is considerable uncertainty in the point estimates (as illustrated with the overlap of the 95% confidence intervals). Differential dialysis mortality among the populations has recently been demonstrated to be modality dependent with Aboriginals on peritoneal dialysis having a higher modality compared to Caucasians [18]. This effect was not observed on hemodialysis.

While reduced access to transplantation is clearly a finding that crosses multiple minority groups in differing regions of the world, in Canada it is the young Aboriginal population in whom this discrepancy is most predominant. We found both deceased and living donor transplants were less likely in Aboriginals. Furthermore in Aboriginals, the adjusted relative rate ratios of living donor transplant were nearly half that of deceased donors (0.32 (95%CI 0.22-0.48) vs. 0.55(95%CI 0.39-0.76)). The reasons behind this reduced rate are often complex involving patient, graft and health delivery-related factors. A report from Alberta, demonstrated Aboriginals were more likely to be in the process of evaluation as opposed to being list as ready or not suitable [19]. Furthermore the median duration for transplant evaluation was 954 days compared to 596 days in Caucasians. In a recent study from Manitoba, Aboriginal potential donors were often excluded due to non-medical reasons, such as loss of contact [6]. Our findings are consistent with previous studies and further demonstrate reduced Aboriginal deceased and living donor transplantation is not limited to the Prairie Provinces alone. Other factors behind the reduced transplant rate may include lower socioeconomic status, language and cultural barriers including mistrust, discrimination, and belief in traditional healing methods [4]. Difficulties in health care delivery such as residing in a rural community and the complexity and time involved in evaluation, follow up, and investigations also significantly contribute [20,21]. Aboriginals have similar rates of referral for renal transplantation but are much more likely to be lost to follow up or not complete the series of steps required to be listed on the active transplant recipient list [19].

Age has been previously identified as an important effect modifier for transplantation in other populations [8,15]. Gender disparities have been well described in the transplant literature as elderly woman are much less likely to receive a renal transplant compared to elderly men [8].

Now that a specific subgroup of the Aboriginal ESRD population, namely the younger individuals, has been identified as the group least likely to receive a renal transplant, race-specific targeted interventions may aid in reducing this disparity. To address the increased length of time potential Aboriginal transplant recipients spend in the post-referral pre-wait list stage, attempts should be made to streamline the workup process as many patients must currently travel long distances, multiple times for various appointments and tests. The implementation of a nationwide program, with guided input from Aboriginal figures who may better understand the potential cultural barriers currently in place, needs to be strongly considered before this problem escalates further. A toolkit prototype designed primarily by Aboriginal people has been developed in New Brunswick, Canada in order to provide culturally-sensitive and relevant information for Aboriginal patients starting on dialysis [22]. Depending on the success of this project, a similar undertaking for the potential transplant population might merit consideration. Education need not only be provided to potential recipients but also to donors as well; given the dwindling supply of deceased donor kidneys, of which most come from the majority Caucasian population and thus may be less suitable for Aboriginal recipients, a concerted effort needs to be made to identify potential living donors and understand the barriers that arise that prevent them from ultimately donating. While these represent possible factors, these beliefs may be individualized and are not necessarily generalizable to the entire Aboriginal population.

There are several potential limitations to this study that should be considered. As this is a retrospective analysis, attempts were made to adjust for confounding although there may still be residual effects unaccounted for, including socioeconomic status, education level and rates of non-adherence. Given the primary outcome measure of time to renal transplantation, we have no knowledge of outcomes post-transplant and whether further racial discrepancies exist at that stage. Studies have shown that Aboriginal transplant recipients have decreased long-term graft survival and also that living donors may be at increased risk post-transplant of hypertension and diabetes [23]. Any potential adverse donor outcomes are particularly important if a focus on increasing the number of Aboriginal living donors is to be taken.

## Conclusion

We found that age is an important effect modifier in determining whether Aboriginals in Canada are likely to undergo renal transplantation. Younger Aboriginals are significantly less likely to receive transplant, a procedure with known improvements in mortality, quality of care and cost effectiveness. Interventions specifically targeting the Aboriginal population are warranted.

## Competing interest

There are no conflicts of interest.

## Authors’ contributions

NT, CR, PK, KY, SP, BH, LS participated in the study design and drafting the manuscript, JM participated in the statistical analysis, SP participated in writing the first draft of the manuscript, MMS participated in drafting the manuscript, study design, study conception and statistical analysis. All authors approved of this manuscript.

## Pre-publication history

The pre-publication history for this paper can be accessed here:

http://www.biomedcentral.com/1471-2369/14/11/prepub

## Supplementary Material

Additional file 1 **Table S1. **Adjusted Cox models for receiving a renal transplant by age groups in Aboriginals. Click here for file
