# Supplementary material for: Young aboriginals are less likely to receive a renal transplant: a Canadian national study
Source: BMC Nephrol. 2013 Jan 14;14:11. doi: 10.1186/1471-2369-14-11 (PMC3558346; doi:10.1186/1471-2369-14-11)
Supplement: Additional file 1 — Table S1. Adjusted Cox models for receiving a renal transplant by age groups in Aboriginals. [file 1471-2369-14-11-S1.doc]

**Supplementary Table 1: Adjusted Cox models for receiving a renal transplant by age groups in Aboriginals.**

| AGE group | Model 1: HR (95% CI), p value | Model 2: HR (95% CI), p value | Model 3: HR (95% CI), p value |
| --- | --- | --- | --- |
|  |  |  |  |
| 18-40 | 0.58(0.46-0.73),P<0.0001 | 0.56(0.45-0.71),P<0.0001 | 0.59(0.46-0.75),P<0.0001 |
| 41-50 | 0.56(0.41-0.76)p<0.0001 | 0.56(0.41-0.77),P<0.0001 | 0.57(0.41-0.79)p=0.001 |
| 51-60 | 0.56(0.42-0.73)p<0.0001 | 0.65(0.49-0.86),P=0.003 | 0.60(0.44-0.81)p=0.001 |
| >60 | 1.01(0.66-1.54)p=0.9 | 1.10(0.72-1.69),p0.7 | 1.08(0.70-1.65)p=0.7 |

Model 1 – sex, BMI, region

Model 2 – co-morbidities

Model 3 – cause of ESRD, serum albumin, distance to centre, dialysis modality, pre-dialysis care

CI confidence interval
